# Signatures of natural selection in abiotic stress-responsive genes of *Solanum chilense*

**DOI:** 10.1098/rsos.171198

**Published:** 2018-01-17

**Authors:** Katharina B. Böndel, Tetyana Nosenko, Wolfgang Stephan

**Affiliations:** Section of Evolutionary Biology, Department of Biology II, Ludwig-Maximilian University of Munich, Planegg-Martinsried, Germany

**Keywords:** abiotic stress, local adaptation, plant population genetics, *Solanum chilense*

## Abstract

Environmental conditions are strong selective forces, which may influence adaptation and speciation. The wild tomato species *Solanum chilense*, native to South America, is exposed to a range of abiotic stress factors. To identify signatures of natural selection and local adaptation, we analysed 16 genes involved in the abiotic stress response and compared the results to a set of reference genes in 23 populations across the entire species range. The abiotic stress-responsive genes are characterized by elevated nonsynonymous nucleotide diversity and divergence. We detected signatures of positive selection in several abiotic stress-responsive genes on both the population and species levels. Local adaptation to abiotic stresses is particularly apparent at the boundary of the species distribution in populations from coastal low-altitude and mountainous high-altitude regions.

## 1. Introduction

Wild tomato species (*Solanum* sect. *Lycopersicon*) are native in South America and occur in diverse habitats, including rainforests in Ecuador, hyperarid regions in the Chilean Atacama Desert, coastal regions and high-altitude regions in the Andes [1,2]. Abiotic environmental conditions contribute to the geographical distribution of these species [3]. Many wild tomato species show distinct morphological adaptations to their natural habitat [2,3]. For instance, *S. chilense*, the wild tomato species that can grow under the driest and coldest conditions [2], is characterized by a deep root system that may allow the utilization of deep groundwater, enhancing drought tolerance [2,3].

Abiotic stress response genes and pathways have been studied extensively in *Arabidopsis* and rice [4,5]. Briefly, receptor or sensory genes receive the stress signal and forward it to transcription factors, e.g. through the plant hormone abscisic acid (ABA)-dependent pathway. Transcription factors or regulatory genes bind to *cis*-acting elements of functional genes located downstream thereby activating their transcription. Although abiotic stress response pathways are not entirely understood in the genus *Solanum*, many abiotic stress-responsive genes have been identified using a range of transcriptomic approaches including cDNA libraries and microarrays. Many of these data were generated for plants exposed to either abiotic stress or ABA [6–8].

As abiotic stress-responsive genes are important to ensure survival, natural selection is expected to influence their evolution. In fact, signatures of natural selection were reported for several abiotic stress-responsive genes in the drought-tolerant wild tomato species *S. chilense* and its closely related sister species *S. peruvianum* [9–12]. Signatures suggesting positive selection were detected in the key transcription factor of the cold-signalling pathway, *ICE1* [11]. Members of transcription factor gene families (*Asr*, ABA/water stress/ripening induced; *CBF*, C-repeat binding factor) evolve under positive directional (*Asr4*), purifying (*Asr1*, *CBF3*) and balancing (*CBF2*) selection [9,10]. The key gene of the ABA biosynthesis, *LeNCED1* (9-*cis*-epoxy-carotenoid dioxygenase), was reported to evolve under purifying selection, while a functional gene, the dehydrin *pLC30-15*, exhibits a haplotypic pattern possibly explained by diversifying selection [12]. These signatures were found on both the species (*Asr1*, *CBF3*, *ICE1*, *LeNCED1*) and the population (*Asr4*, *CBF2*, *pLC30-15*) level, implying either species-wide or population-specific selection, i.e. local adaptation.

In the present study, we focus on the evolution of genes and pathways involved in the abiotic stress response in *S. chilense*. This species is an ideal candidate for adaptation studies as it exhibits a patchy distribution and occurs across a range of environmental conditions including hyperarid desert, cold high-altitude and high soil salinity coastal regions in southern Peru and northern Chile [1,13]. We use information from our previous study on the demography of *S. chilense*, in which we generated a multi-gene/multi-population dataset for abiotic stress-responsive genes (candidate genes) and reference genes [14]. Analyses of population averages revealed a clinal pattern indicating a north–south colonization and a substructure consisting of four population groups. In the present study, we employ standard population genetics to explore whether natural selection is acting upon these genes and whether there are differences between the different populations, i.e. whether we can detect signatures of local adaptation. Furthermore, we investigated if and how natural selection acts on the pathways the genes are organized in. Theory predicts that genes with higher connectivity in a gene network, i.e. with many interacting genes, should be under higher constraint than genes with fewer interactions, and thus are expected to evolve under purifying selection [15]. A recent study on the *Arabidopsis* protein interactome has supported this prediction; it showed that genes acting in the centre of a gene network are under stronger evolutionary constraint than genes acting in the periphery of a gene network [16]. On the other hand, one might also argue that a gene with many interactions has more influence and thus, if altered, might accelerate evolution more than a gene with only few interactions. We find an excess of nonsynonymous single-nucleotide polymorphisms (SNPs) and divergence in the abiotic stress-responsive genes in comparison to the reference genes. Furthermore, we identify genes that evolve under positive directional or balancing selection within populations from the coastal low-altitude region and also from high altitudes, suggesting local adaptation.

## 2. Material and methods

### 2.1. The *Solanum chilense* populations

**Figure 1.**
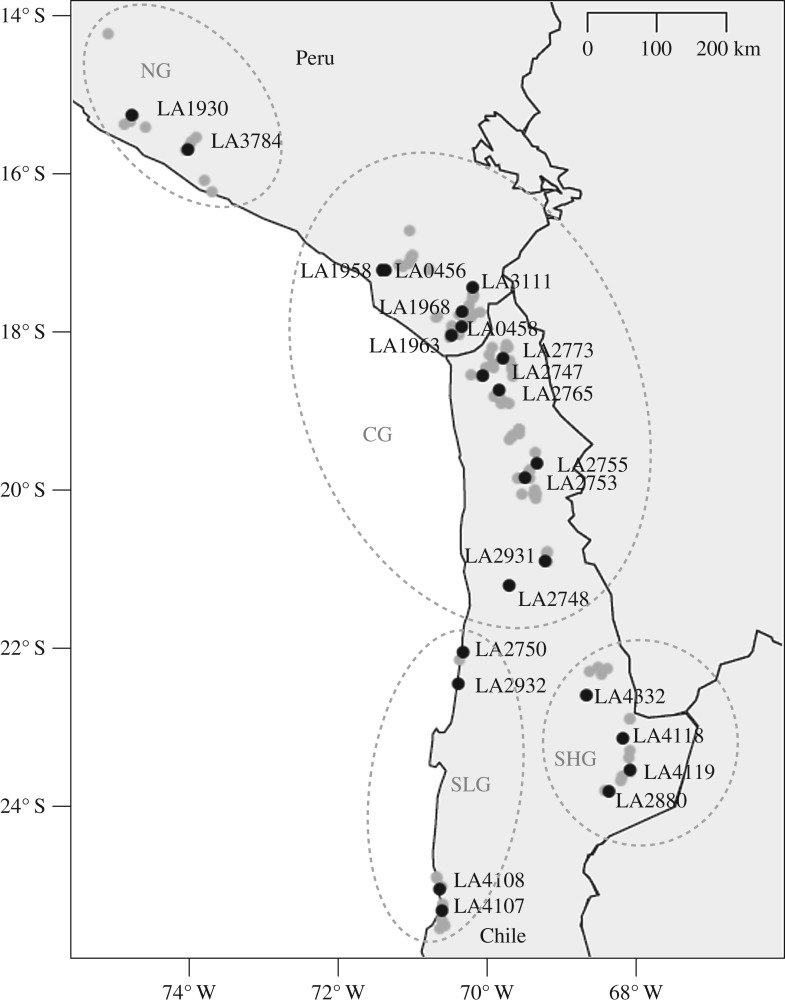
Geographical distribution of the *S. chilense* populations. Map with all *S. chilense* populations sampled by the TGRC (grey circles), the 23 *S. chilense* populations of this study (black circles), and the four population groups that were recently identified (dotted circles; NG, northern group; CG, central group; SLG; southern low-altitude group; SHG, southern high-altitude group) [14].

*Solanum chilense* is a diploid (2*n* = 24), self-incompatible perennial plant, native to a multitude of different habitats in South America ranging from mesic areas in southern Peru to hyperarid areas in the Atacama Desert and the high altitudes in the Andes in northern Chile [1,2,13]. The Tomato Genetics Resource Center (TGRC) at the University of California at Davis (http://tgrc.ucdavis.edu (accessed 16 August 2017)) has a comprehensive collection of wild tomato species and their relatives. Based on available information about sampling locations and population and habitat characteristics, including population sizes and sample sizes, 23 populations (figure 1, electronic supplementary material, table S1) representing the entire species and habitat range were chosen for analyses. One population of *S. ochranthum*, LA2682, was chosen for outgroup comparisons. *Solanum ochranthum* is closely related to the wild tomato clade with an estimated split time of around 5.59 million years [17]. According to our previous study on the species demography these 23 *S. chilense* populations form four population groups: a northern group (NG; including LA1930 and LA3784), a southern high-altitude group (SHG; LA2880, LA4118, LA4119, LA4332), a southern low-altitude group (SLG; LA2750, LA2932, LA4107, LA4108), and a central group (CG; LA0456, LA0458, LA1958, LA1963, LA1968, LA2747, LA2748, LA2753, LA2755, LA2765, LA2773, LA2931, LA3111; figure 1 [14]). The populations of SHG are located in the Andes; the populations of SLG are scattered along the Pacific coast in the southern range of the species distribution. CG covers populations from low and high altitudes. For convenience, we will refer to this grouping throughout this study. For more details on sampling sites, habitat characteristics and breeding procedures at the TGRC, see the electronic supplementary material, table S1, Böndel *et al*. [14] and the TGRC website (http://tgrc.ucdavis.edu (accessed 16 August 2017)).

### 2.2. Choice of genes and sequencing approach

We have recently re-sequenced a set of 30 genes from these 23 *S. chilense* populations in a pooling approach [14]. Sixteen of the genes were previously reported to be involved in the abiotic stress response in *Solanum* sect. *Lycopersicon* or related Solanaceae species like potato or tobacco, and serve as candidate genes for natural selection and local adaptation. They comprise five regulatory genes (*AREB1*, *AREB2*, *JERF1*, *JERF3*, *DREB3*), ten functional genes (*CT208*, *dhn1*, *ER5*, *His1*, *le25*, *LTP*, *pLC30-15*, *TAS14*, *TPP*, *TSW12*) and one sensory gene (*NtC7*; table 1). This classification is common for abiotic stress-responsive genes in plants [5]. Two of the regulatory genes (*AREB1*, *JERF1*) were reported to induce the expression of other genes included in this study [8,19,21], which allows us to link some of our candidate genes into a gene network (electronic supplementary material, figure S1). To our knowledge these are the only direct interactions between our candidate genes that have been reported in *Solanum* sect. *Lycopersicon*. The remaining 14 genes serve as reference genes in this study (electronic supplementary material, table S2). Thirteen reference genes are single-copy cDNA markers [34]; nine of them have been subject to previous studies on genetic variation and demography in wild tomatoes [35–38]. The granule-bound starch synthase I (*GBSSI*) gene was used previously to examine the phylogenetic relationships of wild tomato species [39]. According to our previous analyses, these reference genes are suitable to represent the genomic average of each population [14].

**Table 1.** Abiotic stress-responsive (candidate) genes included in this study.

gene^a^	locus and coordinates^b^	gene description
sensory gene		
* NtC7*	Solyc03g083480; SL2.50ch03:53361485..53362716	receptor-like membrane protein; induced by salt and osmotic stress in *Nicotiana tabacum* [18]
regulatory genes		
* AREB1*	Solyc04g078840; SL2.50ch04:63504038..63500731	AREB/ABF subfamily of basic leucine zipper (bZIP) transcription factor; induced by salt, drought, low temperature and ABA in tomato [8]; induces the expression of *dhn1*, *pLC30-15*, *TAS14*, *le25*, *TSW12* and *TPP* [8,18] (Sol Genomics Network, https://solgenomics.net, accessed 16 August 2017)
* AREB2*	Solyc11g044560; SL2.50ch11:32458669..32463971	AREB/ABF subfamily of basic leucine zipper (bZIP) transcription factor; induced by drought, salt and ABA in tomato, and *AREB1* [19]
* JERF1*	Solyc06g063070; SL2.50ch06:39819912..39817161	ethylene responsive factor (ERF) protein; induced by salt and ABA in tomato [20]; enhances tolerance and growth under salinity and cold temperature in transgenic tobacco [21]; induces expression of *pLC30-15* and *LTP* [21]
* JERF3*	Solyc03g123500; SL2.50ch03:70333120..70335563	ERF protein; induced by cold, salt and ABA in tomato [22]
* DREB3*	Solyc04g072900; SL2.50ch04:59868088..59868882	AP2/EREP transcription factor family; induced by salt, drought and low temperature in tomato [23]
functional genes		
* dhn1* (*ERD10B*)	Solyc02g084840; SL2.50ch02:47921771..47920803	group 2 LEA (dehydrin); induced by low temperature and ABA in potato [24]; induced by *AREB1* [8]
* pLC30-15*	Solyc04g082200; SL2.50ch04:65958867..65957495	group 2 LEA (dehydrin); induced by drought, cold and ABA in *S. chilense* [25,26]; induced by *JERF1* [21]; signatures of local adaptation in *S. chilense* [12]
* TAS14* (*le4*)	Solyc02g084850; SL2.50ch02:47926954..47925819	group 2 LEA (dehydrin); induced by salt, drought and ABA in tomato [6,7]; induced by *AREB1* [8,19]
* ER5*	Solyc01g095140; SL2.50ch01:86522427..86523239	atypical hydrophobic group of LEA; induced by drought and ABA in tomato [27], not upregulated by overexpressing *AREB1* [8]
* le25*	Solyc10g078770; SL2.50ch10:60482744..60481971	group 4 LEA; induced by drought and ABA in tomato [6]; expression in *S. cerevisiae* resulted in higher salt and freezing tolerance [28]; induced by *AREB1* [19]
* LTP* (*NtLTP1*)	Solyc10g075100; SL2.50ch10:58811212..58810582	lipid transfer protein; induced by salt in tomato (GenBank entry DQ073079); induced by *JERF1* [21]
* TSW12*	Solyc10g075110; SL2.50ch10:58833096..58832488	lipid transfer protein; induced by salt in tomato [29]; induced by *AREB1* [19]
* CT208*	Solyc09g064370; SL2.50ch09:61552580..61557226	alcohol dehydrogenase class III; signature of selection in *S. chilense* [30]
* His1* (*le20*)	Solyc02g084240; SL2.50ch02:47397430..47398592	H1 histone gene; induced by drought and ABA in tomato and *S. pennellii* [31,32]; induced by drought in *S. chilense* [32]
* TPP*	Solyc03g083960; SL2.50ch03:53898914..53896143	trehalose-6-phosphate phosphatase; induced by *AREB1* [8,19]

^a^Alternative gene names are given in brackets.

^b^Locus name and coordinates in the genome of the cultivated tomato (*S. lycopersicum* cv. Heinz, SL2.50; Sol Genomics Network, https://solgenomics.net (accessed 16 August 2017 [33]).

The sequence data are publicly available on GenBank (BioProject PRJNA313142, accessions SAMN04524139–SAMN04524161). To obtain this data, genomic DNA of 25 individual plants per population was extracted using the DNeasy Plant Mini Kit (Qiagen). A two-step pooling approach was applied for each population. First, DNA of five individuals was mixed to pre-pools prior to gene amplification by polymerase chain reaction (PCR) using the Phusion® high-fidelity DNA Polymerase kit (Finnzymes, distributed by New England BioLabs, Inc.). Second, the five PCR product pre-pools per gene were brought together during PCR product purification with the MinElute® Gel Extraction Kit (Qiagen), resulting in one PCR product pool per gene. Finally, all 30 PCR product pools per population were mixed in equimolar quantities and concentrated to ≥200 ng µl^−1^ using the Amicon® Ultra-0.5 Centrifugal Filter Devices (Millipore). DNA library construction and high-throughput sequencing (paired-end, 100 bp) on a Genome Sequencer Illumina HiSeq2000 were performed by GATC Biotech AG (Konstanz, Germany). Consequently, this *S. chilense* dataset consists of 575 individual genotypes (25 genotypes from each of the 23 populations) and 1150 alleles (50 alleles from each of the 23 populations) for each gene.

### 2.3. Sequence data assembly and analyses

The *S. chilense* short reads were mapped against sequences from the cultivated tomato genome (*S. lycopersicum* cv. Heinz, release SL2.40) from the SOL genomics network (http://solgenomics.net (accessed 16 August 2017)) using Stampy [40] with the default parameters for paired-end read mapping and a substitution rate of 0.01. We chose Stampy because it showed a higher mapping accuracy than other programs for regions with high genetic variation [41] and gap regions [42]. Three genes (*CT021*, *NtC7* and *TAS14*) were mapped against *S. chilense* sequences because preliminary tests revealed different length in *S. chilense* compared to *S. lycopersicum*. Pileup files were generated with SAMtools [43] and transformed into tables with sequence depth and SNP information for each position. We conducted all data analyses based on these tables. Positions with a sequencing depth below 2000, a base quality and/or mapping quality below 20 were masked and excluded from all analyses. Mutations present in less than 1% of the reads were considered as sequencing errors and therefore excluded from all analyses. Indel polymorphisms were also excluded. The exact numbers of SNPs per gene and population are shown in the electronic supplementary material, table S3.

We previously assessed several statistics for this dataset [14]. We calculated the nucleotide diversity *π*, which is the average number of pairwise nucleotide differences per site [44], and the test statistic Tajima's *D* [45] per gene and population. Tajima's *D* is based on the difference between the Watterson estimator, Θ_W_, and *π*. Tajima's *D* is expected to be 0 under standard neutral expectations. An excess of either low or high frequency polymorphisms leads to a negative Tajima's *D* and indicates purifying or positive directional selection. An excess of intermediate frequency polymorphisms leads to a positive Tajima's *D* and indicates balancing selection. An excess of low frequency polymorphisms and intermediate frequency polymorphisms can further indicate population expansion and population substructure, respectively. We also calculated divergence, *K*, to the outgroup *S. ochranthum*. The *S. ochranthum* sequences were generated previously [14] and are available on GenBank (accessions KY978852–KY978879). We further assessed both *π* and *K* for synonymous (*π*_s_ and *K*_s_) and nonsynonymous (*π*_a_ and *K*_a_) sites separately. We also calculated the pairwise genetic differentiation (*F*_ST_) between each pair of populations for each gene [46].

To address the evolution of gene classes and networks on the species level, we summarized *π*, *π*_s_, *π*_a_, Tajima's *D*, *K*, *K*_s_ and *K*_a_ over all corresponding genes and populations and compared these statistics for (i) reference and candidate genes, (ii) regulatory and functional genes and (iii) subsets of candidate genes that were reported to be linked within networks to reference genes by applying the Wilcoxon rank sum test in R [47]. Each of the two analysed gene networks contains one regulatory and two or five functional genes reported to be induced by the respective regulatory gene: *AREB1* with *dhn1*, *le25*, *pLC30-15*, *TAS14* and *TPP* and *JERF1* with *LTP* and *pLC30-15* (electronic supplementary material, figure S1). We did the same comparisons across populations to investigate whether the observed patterns on the species level are also present in the individual populations.

Then we investigated natural selection and local adaptation by comparing for each population the 16 abiotic stress-responsive genes (candidate genes) with the reference genes, which are assumed to represent the genome average.

We further studied if and how individual SNPs influence the genetic diversity in their immediate neighbourhood; i.e. we were interested in small-scale hitchhiking effects. The motivation for this analysis was that we recently discovered multiple sites characterized by narrow valleys of reduced nucleotide diversity, suggesting small-scale hitchhiking effects, within another abiotic stress-responsive gene, *ICE1*, in *S. chilense* [11]. We followed here the analysis of Lee *et al*. [48] in *Drosophila melanogaster*, because outcrossing tomato species like *S. chilense* show similar characteristics as *D. melanogaster* with regard to recombination rates and diversity levels [35,49,50]. We calculated *π* for 40-bp windows around each SNP. We excluded SNPs when more than three nucleotides within these 40-bp windows were masked and also species-wide fixed differences to *S. ochranthum*, as they likely represent events predating the demographic history of the *S. chilense* populations. We then summarized our results across genes and populations and compared reference genes to candidate genes and the two gene networks, as well as different SNP types (synonymous, nonsynonymous, intronic/intergenic) with each other by applying the Wilcoxon rank sum test.

## 3. Results and discussion

### 3.1. Signatures of adaptive evolution in gene classes and gene networks

**Figure 2.**
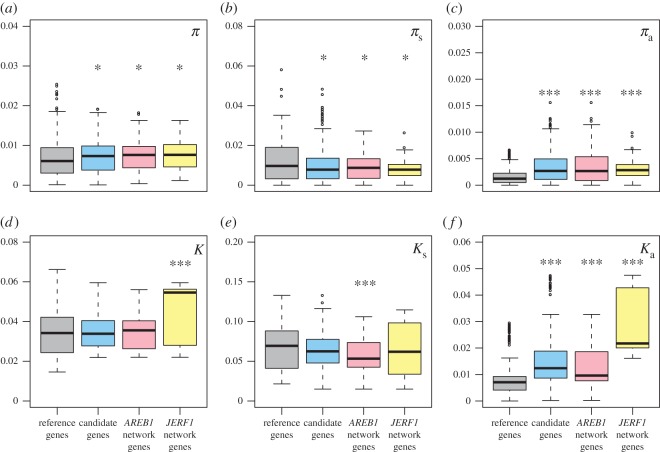
Nucleotide diversity and divergence of the gene classes and networks. The *y*-axis shows values of nucleotide diversity *π* and divergence *K* to *S. ochranthum* summarized over all populations for all sites (*a*,*d*), synonymous sites (*b*,*e*) and nonsynonymous sites (*c*,*f*) for reference genes (grey), candidate genes (blue), the *AREB1* network genes (light pink) and the *JERF1* network genes (yellow). Asterisks indicate significant differences to the reference genes (Wilcoxon rank sum test; *p < 0.05, ***p < 0.001).

We first compared candidate, i.e. abiotic stress-responsive, and reference genes by averaging *π*, *π*_s_, *π*_a_, *K*, *K*_s_, *K*_a_ and Tajima's *D* across all populations. The main finding of this analysis was a highly significant excess of nonsynonymous nucleotide diversity and nonsynonymous divergence in the candidate genes in comparison with the reference genes. Both *π*_a_ and *K*_a_ values were highly significantly elevated (*p* < 0.001) in the candidate genes compared with the reference genes (figure 2). We also found *π* to be significantly elevated (*p* < 0.05) and both *π*_s_ and Tajima's *D* to be significantly reduced (*p* < 0.05) in the candidate relative to the reference genes (figure 2, electronic supplementary material, figure S2). The observed excess of nucleotide diversity, especially at nonsynonymous sites, and nonsynonymous divergence suggests that the candidate genes maintain more adaptive variation and thus may evolve faster. This is in agreement with the observation of an excess of nonsynonymous variation at adaptive genes in *Arabidopsis* [51]. If new environmental conditions are encountered, either because of environmental change or colonization of new habitats, standing genetic variation could become advantageous [52,53]. In fact, in our previous study we discovered possible local adaptation events from standing genetic variation in *S. chilense* [14].

The 16 candidate genes belong to different pathways and different layers of the abiotic stress response (electronic supplementary material, figure S1) and can be classified into sensory, regulatory and functional genes following e.g. Shinozaki & Yamaguchi-Shinozaki [5]. As we have only one sensory gene (*NtC7*), we compared regulatory to functional genes. Functional genes have significantly higher *π*_a_ than regulatory genes (*p* < 0.001; electronic supplementary material, figure S3), suggesting that they contribute most to the candidate versus reference gene *π*_a_ pattern described above. As functional genes are usually located more downstream in the abiotic stress response pathways than regulatory genes, this observation is in agreement with the analyses of the anthocyanin pathway, which showed that upstream genes evolve under stronger constraint than downstream genes [54,55]. This pattern may be explained with a higher connectivity of the upstream genes in gene networks [54,55] and stronger evolutionary constraints acting upon the higher connectivity genes [15].

**Figure 3.**
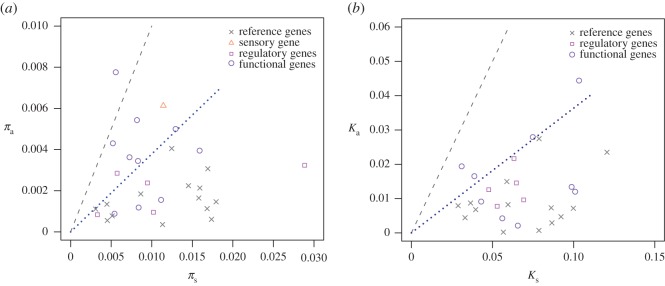
Nonsynonymous and synonymous nucleotide diversity and divergence. (*a*) Nucleotide diversity; the dashed line indicates a *π*_a_/*π*_s_ ratio of 1, the dotted line indicates the upper boundary of the reference genes. (*b*) Divergence to *S. ochranthum*; the dashed line indicates a *K*_a_/*K*_s_ ratio of 1. Gene classes are indicated as follows: grey crosses, reference genes; orange triangle, sensory gene; pink boxes, regulatory genes; purple circles, functional genes.

High ratios of nonsynonymous to synonymous nucleotide diversity (*π*_a_/*π*_s_) or divergence (*K*_a_/*K*_s_) relative to the genomic average are usually considered to indicate adaptive evolution ([56], chapters 7 and 8). Candidate gene averages, especially functional gene averages, are above or closer to a *π*_a_/*π*_s_ ratio of 1 than the averages of the reference genes (figure 3*a*). A similar pattern can be observed for the *K*_a_/*K*_s_ ratios (figure 3*b*). These plots also show variation among genes from the same gene class. The *π*_a_ values of reference and regulatory genes are relatively similar to each other, while the functional genes differ more in *π*_a_ (electronic supplementary material, figure S4*a*). Genes from all classes vary in *K*_a_ (electronic supplementary material, figure S4*b*). The individual genes will be addressed later in this paper.

We also analysed two gene networks in comparison to the reference genes: *AREB1* with *dhn1*, *le25*, *pLC30-15*, *TAS14* and *TPP*, and *JERF1* with *LTP* and *pLC30-15*. Both *AREB1* and *JERF1* were reported to induce the respective regulatory genes ([8,19,21]; SOL Genomics Network, https://solgenomics.net/ (accessed 16 August 2017)). Although additional direct interactions between our candidate genes cannot be excluded, to our knowledge, no other interactions have been reported for *Solanum* sect. *Lycopersicon* so far. Similar to the candidate gene averages, both networks show significantly elevated *π*, reduced *π*_s_ (both *p* < 0.05), and highly elevated (*p* < 0.001) *π*_a_ in comparison to the reference genes (figure 2). This may not be surprising as the networks are subsets of the candidate genes. However, the two networks differ in their divergence patterns. The genes of the *JERF1* network show highly significantly elevated *K* (*p* < 0.001) and the genes of the *AREB1* network have significantly decreased *K*_s_ (*p* < 0.05). Like the candidate genes, both have significantly elevated *K*_a_ (*p* < 0.001), this being especially profound for the *JERF1* network genes. The transcription factor *JERF1* and the two genes downstream of *JERF1*, the dehydrin *pLC30-15* and the lipid transfer protein *LTP*, are among the four genes with highest *K*_a_ of all the candidate genes (electronic supplementary material, figure S4*b*), suggesting that this network may be undergoing adaptive evolution in *S. chilense*.

Furthermore, we calculated the aforementioned statistics for each population separately to investigate if the pattern observed on the species level is also present in the individual populations (electronic supplementary material, tables S4–S10). In all populations, averages of *π*_a_, *K* and *K*_a_ are higher for the candidate than for the reference genes; the same holds for *K* and *K*_a_ for the *JERF1* network genes and for *π*_a_ and *K*_a_ for the *AREB1* network genes, while *K* and *K*_s_ are lower for the *AREB1* network genes. The functional genes in comparison to the regulatory genes show higher averages of *π*_a_, *K*_s_ and *K*_a_ in all populations. All other statistics do not show a consistent pattern across populations in the respective gene class or network comparisons. The fact that the patterns observed on the species level are also largely present across populations indicates that the north–south colonization [14] had no major impact on the evolution of gene classes and networks.

### 3.2. Patterns of genetic diversity in the immediate neighbourhood of single-nucleotide polymorphisms

**Figure 4.**
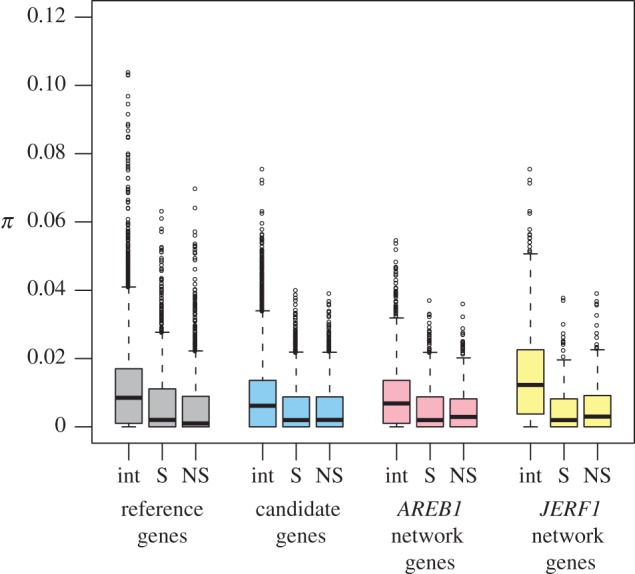
Patterns of nucleotide diversity surrounding SNPs. Values of *π* for 40-bp windows around intronic/intergenic (int), synonymous (S) and nonsynonymous (NS) SNPs in reference genes (grey), candidate genes (blue) and genes of the *AREB1* (light pink) and *JERF1* (yellow) networks.

We have recently discovered elevated nucleotide diversity and Tajima's *D* in the transcription factor *ICE1* in *S. chilense*, which could be explained by interactions of several positively selected sites within the gene [11]. Since we also observed elevated nucleotide diversity, especially at nonsynonymous sites, in the present dataset, we decided to investigate the patterns around individual SNPs to assess the possibility of interactions between several positively selected variants. This analysis revealed that synonymous and nonsynonymous SNPs, i.e. exonic SNPs, show reduced *π* in the immediate neighbourhood in comparison to intronic and intergenic SNPs (figure 4). This reduction is highly significant (*p* < 0.001) in reference genes, candidate genes and also in the genes of the two networks. Wilcoxon tests comparing reference genes to candidate genes and reference genes to each of the two networks revealed also significant differences in *π* for each of the three SNP types (intronic/intergenic, synonymous and nonsynonymous). Furthermore, the reduction of *π* around nonsynonymous SNPs in the two networks appears to be less severe than in all candidate genes. These different degrees of reduction of genetic diversity around nonsynonymous SNPs may be explained by different strengths of selection acting on the candidate genes. Interaction between selected variants may lead to evolutionary traffic [57], which—in turn—is expected to reduce the strength of selection and increase nucleotide diversity between and around selected sites [58]. According to the results of our analyses, the frequency of the SNPs does not seem to greatly influence *π* in the immediate SNP neighbourhood in both candidate and reference genes although the scatter of *π* values is larger in the neighbourhood of SNPs fixed within populations than in the neighbourhood of SNPs in intermediate to high frequencies (electronic supplementary material, figure S5).

### 3.3. Species-wide signatures of adaptation

Analyses of individual genes on the population level identified several abiotic stress-responsive genes that show consistent trends across most populations, which might indicate that similar selective forces are acting upon these genes across the species distribution, i.e. across various environments. Four of these genes, the transcription factors *AREB1* and *AREB2*, the dehydrin *dhn1*, and the alcohol dehydrogenase *CT208*, are characterized by rather low nucleotide diversity, especially at nonsynonymous sites, in comparison to the reference gene averages (electronic supplementary material, tables S4–S6). Further, *K* is reduced in *dhn1*, *K*_s_ in *AREB1*, *AREB2* and *dhn1* and *K*_a_ in *dhn1* and *CT208* (electronic supplementary material, tables S8–S10). This may suggest that these genes are rather conserved in *S. chilense*. However, a selective sweep scenario has been hypothesized previously for the alcohol dehydrogenase *CT208* in *S. chilense* previously, which also agrees with the low nucleotide diversity observed in our study. Arunyawat *et al.*'s [30] main finding to support this hypothesis was a haplotype that decreased in frequency from south to north. Our analysis of pairwise genetic differentiation (*F*_ST_) between populations shows relatively low *F*_ST_ between populations from the southern and/or central regions and elevated *F*_ST_ between the two most northern populations and populations from either central or southern regions (electronic supplementary material, figure S9*b*), which would agree with the haplotype pattern detected by Arunyawat *et al*. [30] and support the hypothesis of a selective sweep scenario across a large part of the species distribution.

Other genes are characterized by rather high levels of nucleotide diversity and/or divergence in comparison to the reference gene averages (electronic supplementary material, tables S4–S6, S8–S10): the sensory gene *NtC7*, the transcription factors *JERF1* and *DREB3*, the *lea* genes *ER5* and *le25*, and the two lipid transfer protein encoding genes *LTP* and *TSW12*. *NtC7* and *JERF1* are further characterized by elevated Tajima's *D* values (electronic supplementary material, table S7). Elevated Tajima's *D*, i.e. an excess of intermediate frequency polymorphisms, may be indicative of balancing selection. The NtC7 protein was reported to have a receptor-like function and to show structural similarities to tomato Cf-9, which is involved in the biotic stress response [18]. Originally, *NtC7* was identified as a wound-response gene [59] and shown to be induced by osmotic stresses [18]. These findings could imply that *NtC7* plays a role in both abiotic and biotic stress response. Balancing selection acting upon biotic stress-responsive genes is not uncommon in wild tomatoes [60,61]. Transcription factors could be involved in crosstalk between abiotic and biotic pathways [62]. The signature of balancing selection for *JERF1* could be explained by an involvement of this gene in different pathways in tomato as suggested by expression analyses [20]. The excess of (nonsynonymous) nucleotide diversity and divergence, which is observed for most abiotic stress-responsive genes, indicates evidence for adaptive evolution as discussed in §3.1.

### 3.4. Signatures of local adaptation

Some abiotic stress-responsive genes show signatures of selection across certain parts of the species range that are characterized by specific environments and may thus suggest local adaptation. For example, consistent signatures of positive selection are found in several genes of the SLG populations LA2750, LA2932, LA4107 and LA4108, whose habitat is influenced by the Pacific coast and the Atacama Desert. The SLG populations show reduced *F*_ST_ for the genes *dhn1*, *le25*, and *JERF3* in comparison to the reference gene averages (electronic supplementary material, figures S7 and S8). These three genes are known to be involved in the salt stress response. *JERF3* is induced by salt stress [61]. The expression of *dhn1* and *le25* is induced by the transcription factor *AREB1*, which is induced by salt stress [8,19]. *AREB1* does not show reduced genetic differentiation within this group (electronic supplementary material, figure S6*b*). However, we previously identified three outlier SNPs for positive selection in *AREB1* and all of them are either in high frequency or fixed in the SLG populations [14]. A positive selection scenario for *AREB1* in the SLG populations is further supported by reduced Tajima's *D* values in comparison to the reference genes (electronic supplementary material, table S7). The Tajima's *D* values of *dhn1*, *le25* and *JERF3* are also reduced in the SLG populations (electronic supplementary material, table S7). However, the low nucleotide diversity and divergence of *dhn1* may suggest purifying instead of positive selection (see §3.3). *JERF3* and *le25*, on the other hand, show elevated *K*_a_ in SLG in comparison to the respective reference gene averages (electronic supplementary material, table S10) which is in agreement with positive selection. These findings imply that salinity may have been an important selective force acting upon these genes.

Different selection regimes seem to be prevalent in high-altitude populations. The high-altitude habitats of the CG populations are characterized by low temperatures; the SHG populations are exposed to low temperatures and also drought. *JERF3* has reduced Tajima's *D* and low *F*_ST_ in comparison to the reference gene averages in all SHG populations (electronic supplementary material, table S7 and figure S7*a*), which suggests positive selection and is in agreement with three previously identified outlier SNPs [14]. As *JERF3* is induced by cold stress [22], adaptation to low temperatures may be possible. However, because *JERF3* also showed signatures of positive selection in the SLG populations, this could imply that the observed pattern is specific to the southern range of the species distribution and must not necessarily be due to selection. However, we find that *F*_ST_ between the SLG and SHG populations is not reduced for *JERF3* (electronic supplementary material, figure S7*a*). This suggests that selection in these two population groups may act on different molecular variants.

The dehydrin *pLC30-15* is characterized by reduced Tajima's *D*, elevated *K*_a_ and *F*_ST_ in comparison to the reference gene averages in one high-altitude population from the northern CG range, namely LA3111 (electronic supplementary material, tables S7, S10 and figure S7*d*). These findings and one previously identified outlier SNP [14] may suggest that *pLC30-15* evolves under positive selection in LA3111. Previously *pLC30-15* was reported to evolve under diversifying selection in another *S. chilense* population [12] from the northern range of the distribution of the CG populations. Also other populations of this area, e.g. LA0456, LA1958 or LA1968, have reduced Tajima's *D* and elevated *K*_a_ for *pLC30-15* in comparison to the respective reference gene averages (electronic supplementary material, tables S7 and S10), suggesting that *pLC30-15* may be under positive selection in this area. Since the strongest signature is observed in LA3111, which exists in one of the coldest habitats, this could imply that *pLC30-15* is involved in adaptation to low temperatures. In fact, *pLC30-15* was reported to be upregulated under low-temperature stress [25].

The populations LA2880 and LA4118 (both SHG) are characterized by elevated Tajima's *D* and also by elevated *π* for the transcription factor *AREB1* and the dehydrin *TAS14* compared to the respective reference gene averages (electronic supplementary material, tables S4 and S7), which may suggest that balancing selection might be acting on these two genes in these two high-altitude populations. Furthermore, *TAS14* has elevated *π*_a_ in both populations (electronic supplementary material, table S6). Interestingly, *TAS14* is induced by *AREB1* [8,19] indicating that selection may be acting on both genes in the same way. The occurrence of balancing selection may be unexpected for abiotic stress-responsive genes as it has primarily been found for genes involved in the biotic stress response [63]. As the SHG populations are confronted with low temperatures, drought and partly also with high salt concentrations in the soil [1] and both genes were reported to be involved in more than one abiotic stress response [6–8], varying climatic and/or environmental conditions could explain the balancing selection signature. For example, *CBF2*, a transcription factor from the cold-signalling pathway, was reported to evolve under balancing selection in wild tomato species [10]. Expression analysis revealed that one allele was induced by cold stress and another one by drought stress. Such a scenario may be possible for *AREB1* and *TAS14* as well. Recently, patterns consistent with balancing selection were also reported for *ICE1*, the key gene of the cold-signalling pathway [11]. In this case, however, balancing selection was excluded and the observed pattern was explained by interactions of positively selected SNPs leading to intermediate frequency polymorphisms between the selected sites [58]. Such a scenario may also be possible. Our results as well as the two aforementioned studies indicate that signatures consistent with balancing selection may be more frequent in abiotic stress-responsive genes than expected and that this may be an interesting direction for future research on the evolution of abiotic stress-responsive genes.

## 4. Conclusion

The wild tomato species *S. chilense* is exposed to extremely variable and extreme environmental conditions. It is, therefore, reasonable to assume that frequent adaptations have occurred in the past. In this study, we focused on a set of 16 abiotic stress-responsive genes. Our analyses revealed elevated nucleotide diversity and divergence, especially at nonsynonymous sites, which may suggest that these genes have evolved under positive selection. We also found interesting results for several individual genes, which may provide a basis for future studies. Some genes (e.g. *NtC7*, *dhn1*) were detected to have patterns that are consistent across the majority of populations, which may indicate that selection pressures are homogeneous across most of the species range. However, we also identified genes (e.g. *AREB1*, *JERF3*, *pLC30-15*) with signatures specific to certain environments, which may suggest local adaptation. Interestingly, two genes, *JERF3* and *AREB1*, may be involved in different adaptation processes because selection seems to be acting on different molecular variants within the genes: adaptation to coastal as well as to high-altitude conditions. Another interesting finding is that two interacting genes, *AREB1* and *TAS14*, show similar signatures in two populations. Some of our candidate genes are members of the same gene families (e.g. *JERF1* and *JERF3*) and our results suggest different evolutionary histories for the individual paralogues. We did not address gene family evolution further here but anticipate that wild tomatoes are suitable for future studies in this direction. Although our dataset is relatively limited with only 16 genes, it provides a first step to the evolutionary genetic analyses of whole abiotic stress pathways in wild tomatoes.

## Supplementary Material

Supplementary tables and figures

## Supplementary Material

Supplementary tables

## Data accessibility

The dataset used in this study is available on GenBank (pooled *S. chilense* sequences: accessions SAMN04524139–SAMN04524161, BioProject PRJNA313142; *S. ochranthum* sequences accessions KY978852–KY978879).
