# Supplementary material for: Signatures of natural selection in abiotic stress-responsive genes of Solanum chilense
Source: R Soc Open Sci. 2018 Jan 17;5(1):171198. doi: 10.1098/rsos.171198 (PMC5792908; doi:10.1098/rsos.171198)
Supplement: Supplementary tables and figures [file rsos171198supp1.pdf]

Supplementary online materials for

Signatures of natural selection in abiotic stress-responsive genes of *Solanum chilense*

Katharina B. Böndel, Tetyana Nosenko, Wolfgang Stephan

**Supplementary tables**		**2**
Table S1	Geographic and climatic information of the wild tomato populations.	2
Table S2	List of the 14 reference genes.	3
**Supplementary figures**		**4**
Figure S1	Schematic overview over the candidate genes.	4
Figure S2	Tajima's *D* of gene classes and single genes.	5
Figure S3	Nucleotide diversity and divergence of regulatory and functional genes.	6
Figure S4	Nonsynonymous nucleotide diversity and divergence.	7
Figure S5	SNP frequency vs. nucleotide diversity in 40-bp windows.	8
Figure S6	Pairwise genetic differentiation of *NtC7*, *AREB1*, *AREB2*, and *JERF1* in comparison to the reference gene averages.	9
Figure S7	Pairwise genetic differentiation of *JERF3*, *DREB3*, *dhn1*, and *pLC30-15* in comparison to the reference gene averages.	10
Figure S8	Pairwise genetic differentiation of *TAS14*, *ER5*, *le25*, and *LTP* in comparison to the reference gene averages.	11
Figure S9	Pairwise genetic differentiation of *TSW12*, *CT208*, *His1*, and *TPP* in comparison to the reference gene averages.	12
**References**		**13**

Supplementary tables

Table S1. Geographic and climatic information of the wild tomato populations.

Population[a]	Province/Department, Country[b]	Coordinates (latitude, longitude)[b]	Elevation [m a.s.l.][b]	Mean annual temperature [°C][c]	Annual precipitation [mm][c]
Solanum chilense					
Northern group (NG)					
LA1930	Arequipa, Peru	15° 17' 30" S, 74° 36' 0" W	500	19.6	6
LA3784	Arequipa, Peru	15° 43' 52" S, 73° 51' 2" W	1100	17.3	18
Central group (CG)					
LA0456	Moquegua, Peru	17° 15' 0" S, 71° 12' 0" W	200	16.9	8
LA1958	Moquegua, Peru	17° 15' 0" S, 71° 15' 0" W	1250	17.1	6
LA3111	Tacna, Peru	17° 28' 0" S, 70° 2' 0" W	3070	11.6	121
LA1968	Tacna, Peru	17° 45' 42" S, 70° 10' 35" W	1680	15.8	18
LA0458	Tacna, Peru	17° 58' 0" S, 70° 11' 0" W	800	17.5	18
LA1963	Tacna, Peru	18° 4' 0" S, 70° 19' 0" W	200	18.2	11
LA2773	Arica and Parinacota, Chile	18° 22' 0" S, 69° 38' 0" W	3400	10.3	142
LA2747	Arica and Parinacota, Chile	18° 35' 0" S, 69° 54' 0" W	800	16.5	9
LA2765	Arica and Parinacota, Chile	18° 46' 0" S, 69° 41' 0" W	2400	12.3	51
LA2755	Tarapaca, Chile	19° 41' 3" S, 69° 10' 52" W	3200	7.3	103
LA2753	Tarapaca, Chile	19° 51' 23" S, 69° 20' 14" W	1650	14.1	17
LA2931	Tarapaca, Chile	20° 55' 0" S, 69° 4' 0" W	2275	12.3	18
LA2748	Tarapaca, Chile	21° 12' 48" S, 69° 32' 52" W	800	17.7	1
Southern high altitude group (SHG)					
LA4332	Antofagasta, Chile	22° 36' 32" S, 68° 31' 19" W	2968	10.5	29
LA4118	Antofagasta, Chile	23° 9' 27" S, 68° 2' 6" W	2440	13.5	48
LA4119	Antofagasta, Chile	23° 33' 14" S, 67° 56' 2" W	2980	11.4	40
LA2880	Antofagasta, Chile	23° 49' 0" S, 68° 13' 0" W	2500	13.9	36
Southern low altitude group (SLG)					
LA2750	Antofagasta, Chile	22° 4' 13" S, 70° 9' 47" W	300	18.8	2
LA2932	Antofagasta, Chile	22° 28' 5" S, 70° 13' 30" W	300-400	18.5	2
LA4108	Antofagasta, Chile	25° 3' 9" S, 70° 28' 33" W	80	18.4	18
LA4107	Antofagasta, Chile	25° 19' 8" S, 70° 26' 46" W	86	18.2	22
Solanum ochranthum					
LA2682	Cusco, Peru	13° 38' 2" S, 72° 14' 11" W	2500	13.4	740

[a]*S. chilense* populations within groups sorted from north to south.
[b]Geographic data from the TGRC website (http://tgrc.ucdavis.edu, last accessed 16 Aug 2017).
[c]Climatic data from the WorldClim database (http://www.worldclim.org, last accessed 16 Aug 2017).

Table S2. List of the 14 reference genes.

**Gene**	**Locus**[a]	**Location**[a]	**Description**
CT021	Solyc06g035580	SL2.50ch06:24603412..24597492	choline dehydrogenase
CT066	Solyc10g054440	SL2.50ch10:55419938..55417815	arginine decarboxylase
CT093	Solyc05g010420	SL2.50ch05:4655463..4654381	S-adenosylmethionine decarboxylase
CT114	Solyc07g066600	SL2.50ch07:67985084..67982104	phosphoglycerate kinase
CT143	Solyc09g009040	SL2.50ch09:2385547..2393745	delta(14)-sterol reductase
CT166	Solyc02g083810	SL2.50ch02:47072154..47068869	ferredoxin--NADP reductase
CT179	Solyc03g120470	SL2.50ch03:68860050..68850810	aquaporin
CT182	Solyc11g011960	SL2.50ch11:4919067..4912805	UTP-glucose 1 phosphate uridylyltransferase
CT189	Solyc12g039120	SL2.50ch12:45441991..45443480	40S ribosomal protein S19-like
CT192	Solyc04g015130	SL2.50ch04:5295967..5292846	ribosomal protein S6 kinase alpha-3
CT198	Solyc09g082650	SL2.50ch09:68367293..68372036	acireductone dioxygenase
CT251	Solyc02g036370	SL2.50ch02:30604296..30601627	MYB transcription factor
CT268	Solyc01g007130	SL2.50ch01:1677070..1680707	receptor like kinase
GBSSI	Solyc08g083320	SL2.50ch08:65819075..65815703	granule-bound starch synthase

[a]Locus name and coordinates in the genome of the cultivated tomato (*S. lycopersicum* cv. Heinz, SL2.50, Sol Genomics Network, https.//solgenomics.net, last accessed 16 Aug 2017 [1]).

Supplementary figures

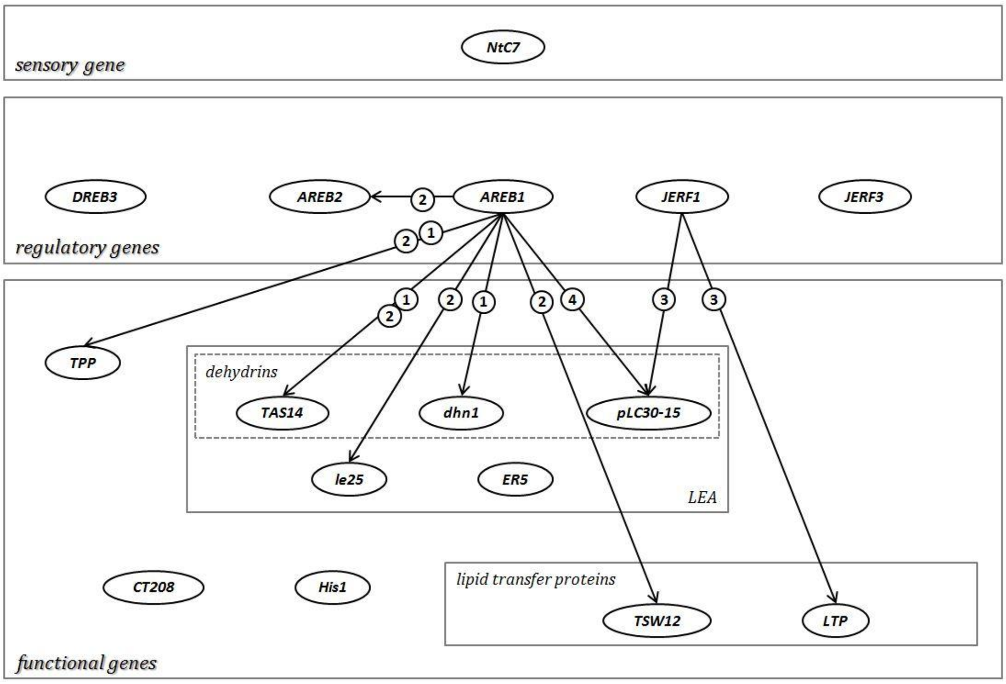

Figure S1. Schematic overview over the candidate genes. Candidate genes (table 1) were classified as sensory, regulatory or functional genes following Shinozaki and Yamaguchi-Shinozaki [2]. Boxes indicate the layers of the stress response (sensory genes, regulatory genes, functional genes) or groups within the functional genes (dehydrins, LEA, lipid transfer proteins). Two transcription factors were reported to induce the expression of other genes: 1 [3], 2 [4], 3 [5], and 4 SOL genomics network (http://solgenomics.net/).

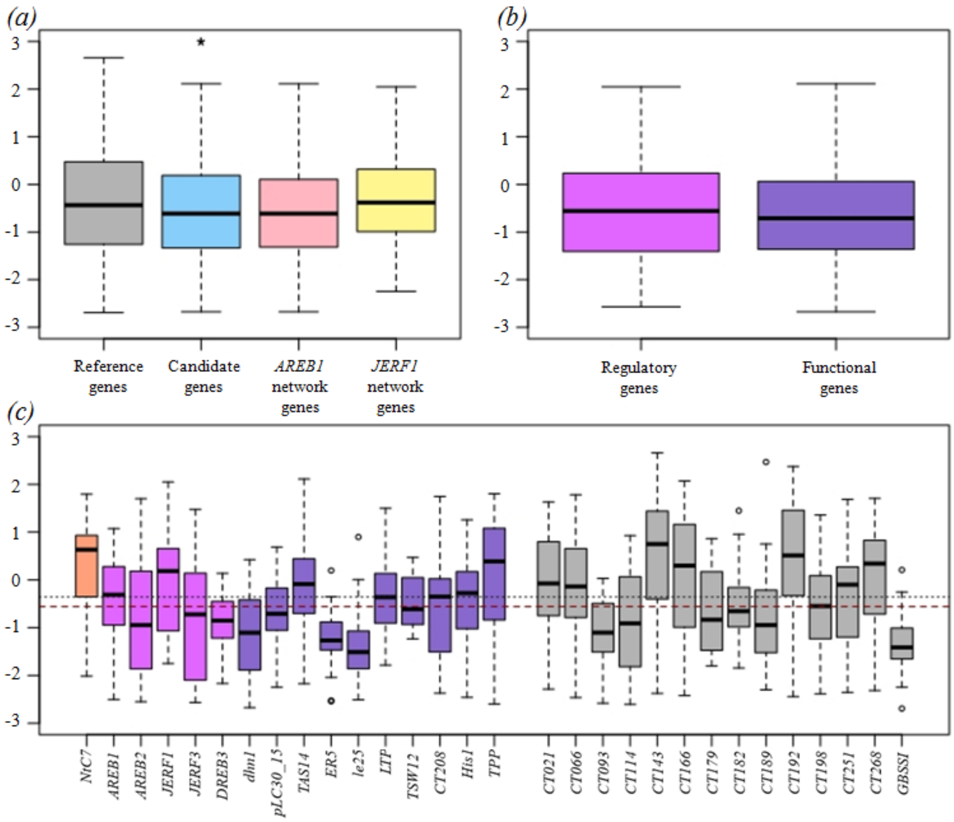

Figure S2. Tajima's *D* of gene classes and single genes. Tajima's *D* summarized over all populations for *(a)* reference (grey) and candidate (blue) genes and genes of *AREB1* (light pink) and *JERF1* (yellow) networks, and *(b)* regulatory (pink) and functional (purple) genes. Star indicates significant differences between classes (Wilcoxon rank sum test; * p-value < 0.05). *(c)* Tajima's *D* summarized over all populations for each gene. Dotted line shows the average Tajima's *D* of the reference genes, red dashed line shows the average Tajima's *D* of the candidate genes. From left to right: sensory gene (orange; *NtC7*), regulatory genes (pink; *AREB1 – DREB3*), functional genes (purple; *dhn1 – TPP*), reference genes (grey; *CT021 – GBSSI*).

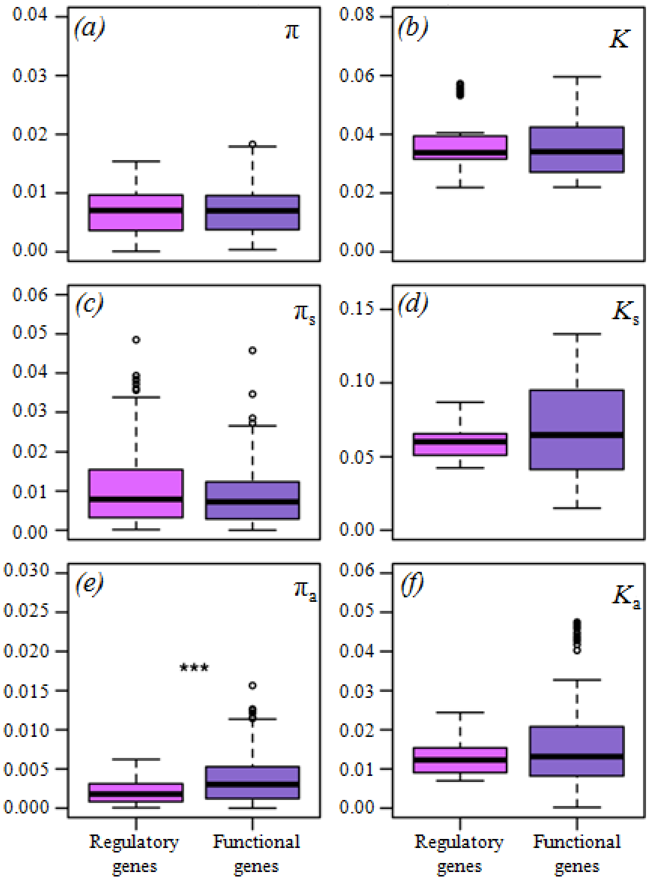

Figure S3. Nucleotide diversity and divergence of regulatory and functional genes. Nucleotide diversity, π, and divergence, *K*, to *S. ochranthum* summarized over all populations for all sites *(a, b)*, synonymous sites *(c, d)* and nonsynonymous sites *(e, f)* for regulatory (pink) and functional (purple) genes. Stars indicate significant differences between the classes (Wilcoxon rank sum test; *** p-value < 0.001).

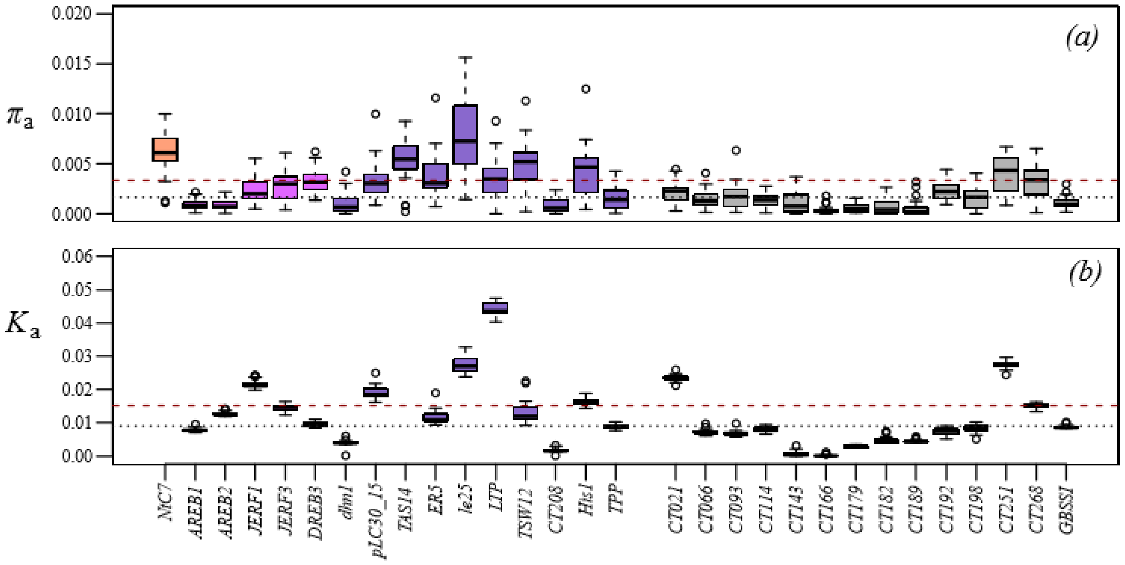

Figure S4. Nonsynonymous nucleotide diversity and divergence. Nonsynonymous nucleotide diversity *(a)* and nonsynonymous divergence to *S. ochranthum* *(b)* summarized over all populations for each gene. Dotted lines show average of the reference genes, red dashed lines show average of the candidate genes. From left to right: sensory gene (orange; *NtC7*), regulatory genes (pink; *AREB1* – *DREB3*), functional genes (purple; *dhn1* – *TPP*), reference genes (grey; *CT021* – *GBSSI*). Note that *S. ochranthum* sequences are not available for *NtC7* and *TAS14*.

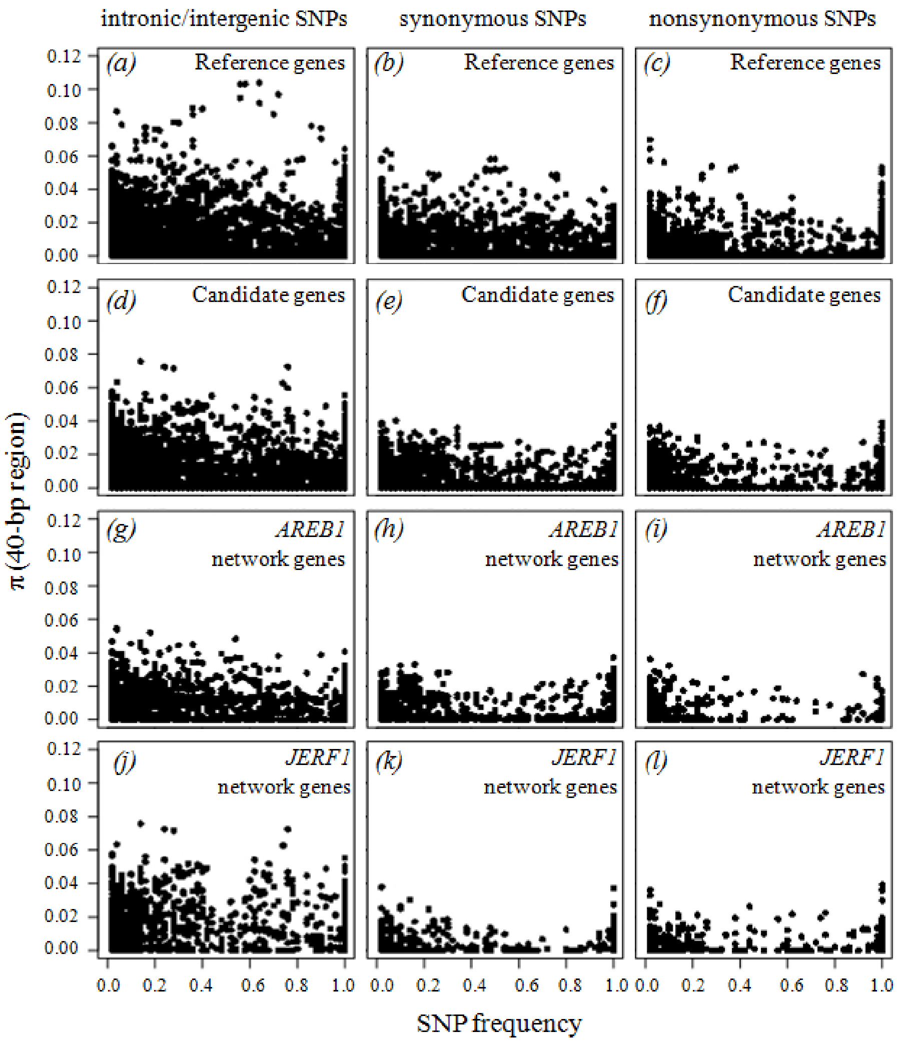

Figure S5. SNP frequency vs. nucleotide diversity in 40-bp windows. Nucleotide diversity, π, in 40-bp windows around each individual SNP was plotted against the frequency of this SNP. Reference genes *(a-c)*, candidate genes *(d-f)*, genes from the *AREB1* network *(g-i)* and from the *JERF1* network *(j-l)* for intronic/intergenic *(a, d, g, j)*, synonymous *(b, e, h, k)*, and nonsynonymous *(c, f, i, l)* SNPs.

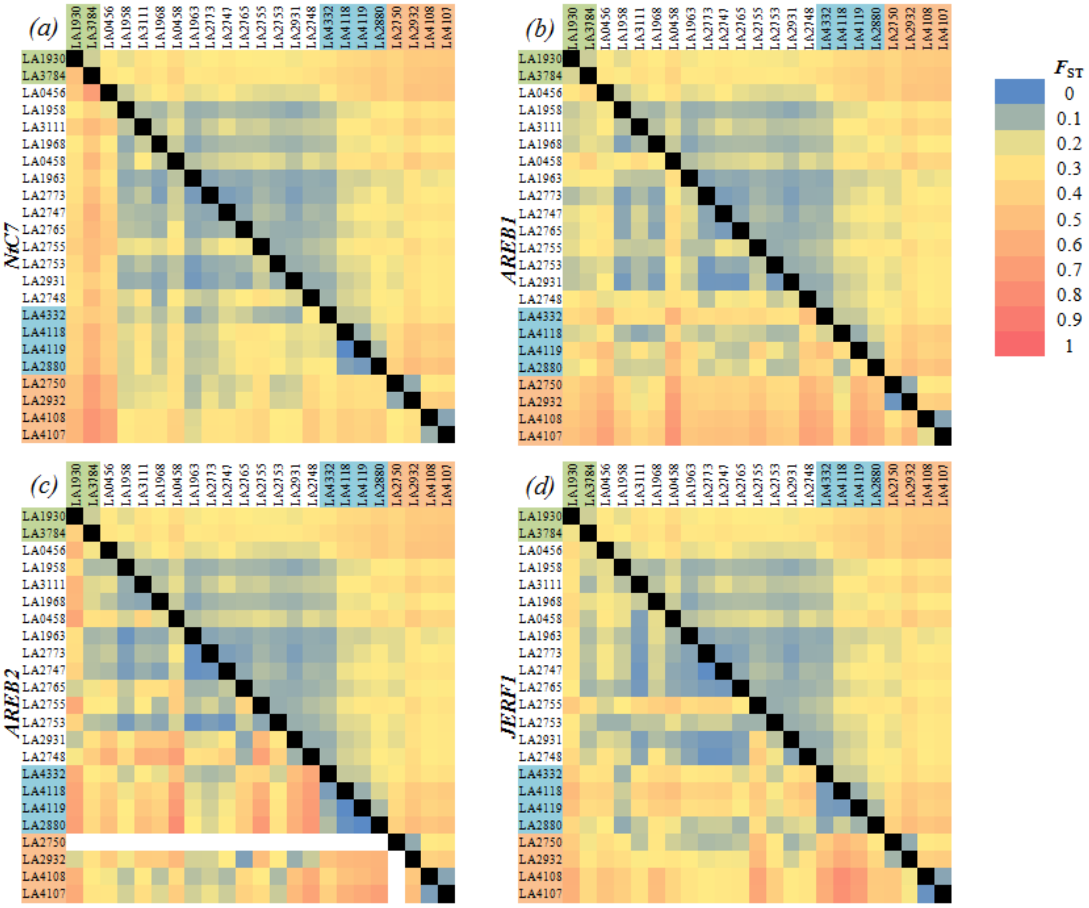

Figure S6. Pairwise genetic differentiation of *NtC7*, *AREB1*, *AREB2*, and *JERF1* in comparison to the reference gene averages. Pairwise F_{ST} for *(a) NtC7*, *(b) AREB1*, *(c) AREB2*, and *(d) JERF1* below the diagonal and average pairwise F_{ST} of the reference genes above the diagonal. F_{ST} values are represented in colour from blue (F_{ST} = 0) to red (F_{ST} = 1). Populations are sorted top-to-bottom and left-to-right according to population group (green: northern group (NG); white: central group (CG); blue: southern high altitude group (SHG); orange: southern low altitude group (SLG)) and latitude (north to south) within each group.

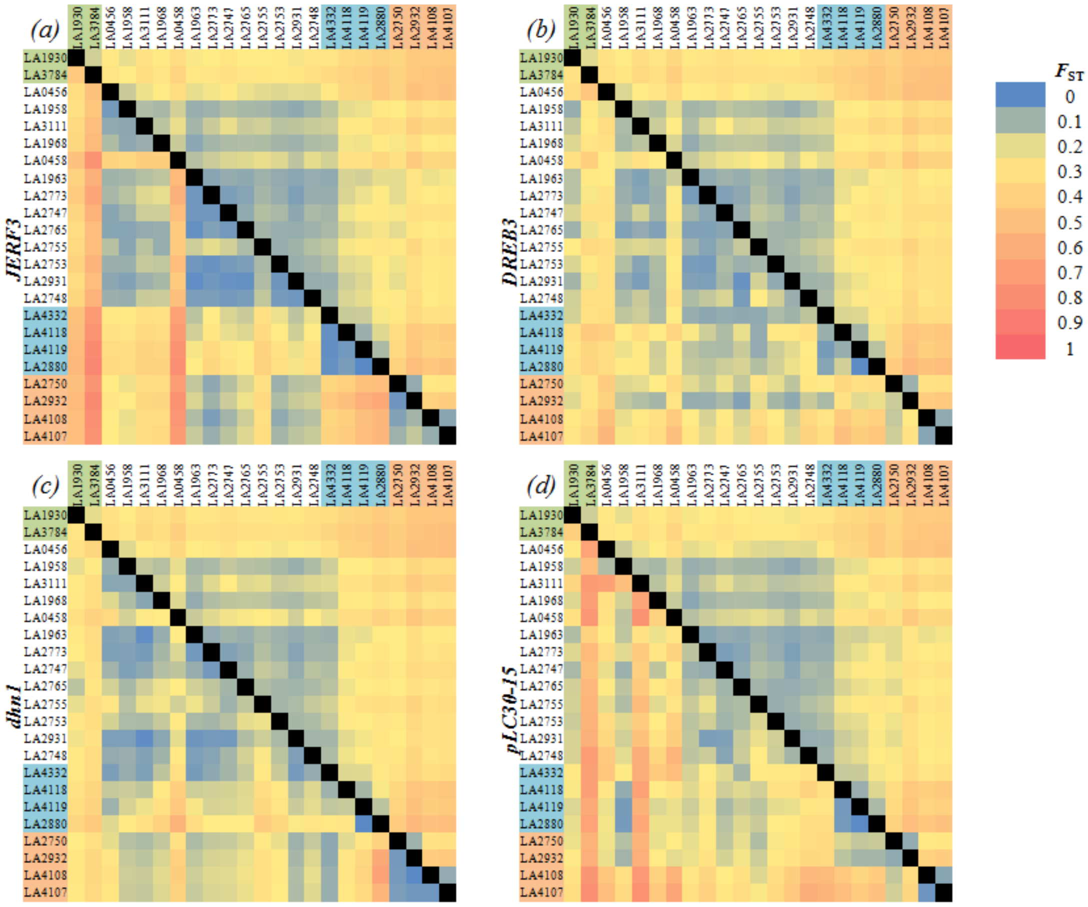

Figure S7. Pairwise genetic differentiation of *JERF3*, *DREB3*, *dhn1*, and *pLC30-15* in comparison to the reference gene averages. Pairwise F_{ST} for *(a) JERF3*, *(b) DREB3*, *(c) dhn1*, and *(d) pLC30-15* below the diagonal and average pairwise F_{ST} of the reference genes above the diagonal. F_{ST} values are represented in colour from blue (F_{ST} = 0) to red (F_{ST} = 1). Populations are sorted as in figure S6.

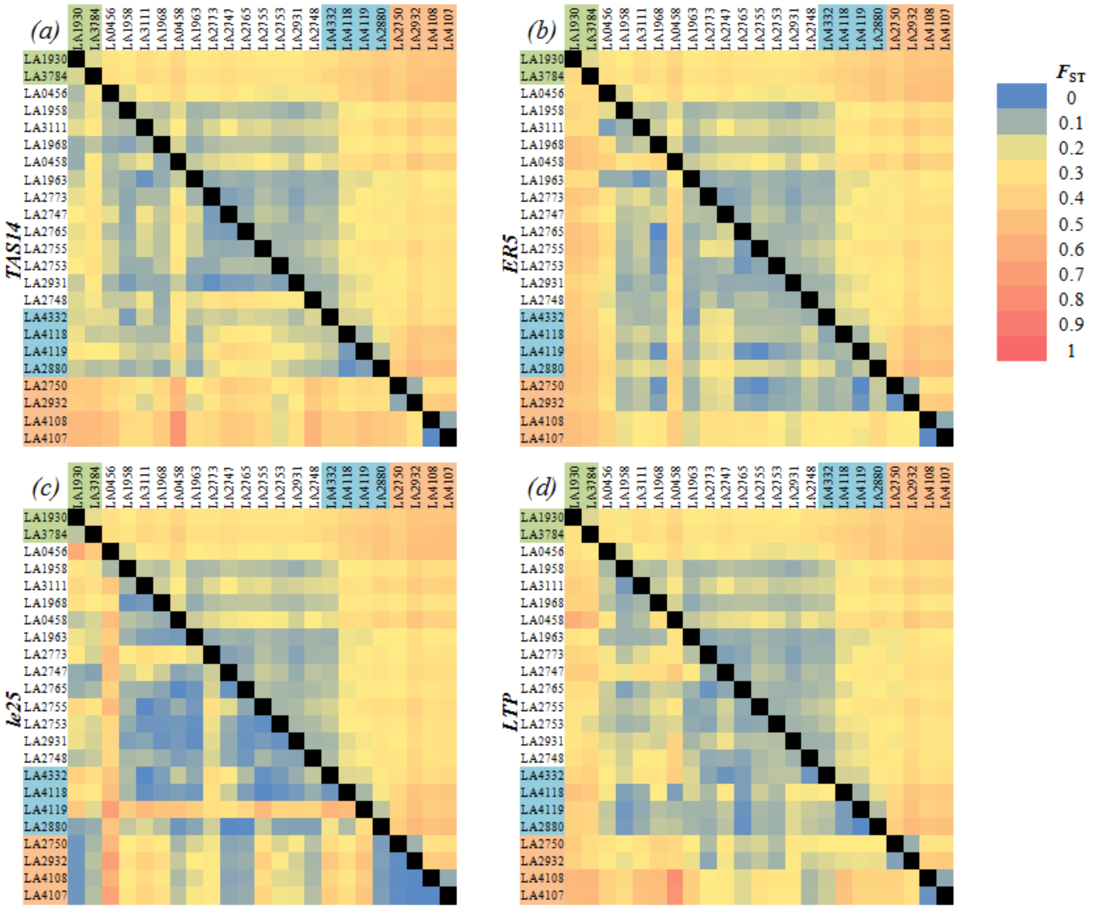

Figure S8. Pairwise genetic differentiation of *TAS14*, *ER5*, *le25*, and *LTP* in comparison to the reference gene averages. Pairwise F_{ST} for *(a) TAS14*, *(b) ER5*, *(c) le25*, and *(d) LTP1* below the diagonal and average pairwise F_{ST} of the reference genes above the diagonal. F_{ST} values are represented in colour from blue (F_{ST} = 0) to red (F_{ST} = 1). Populations are sorted as in figure S6.

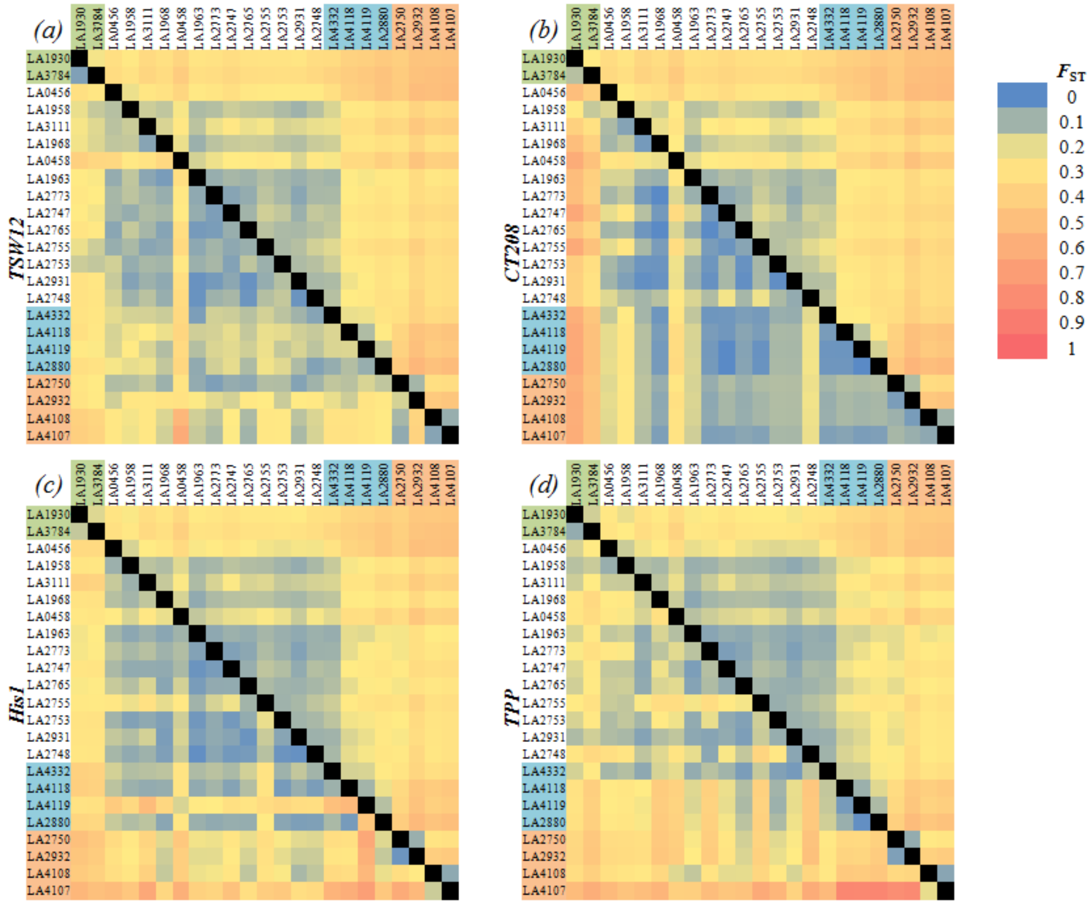

Figure S9. Pairwise genetic differentiation of *TSW12*, *CT208*, *His1*, and *TPP* in comparison to the reference gene averages. Pairwise F_{ST} for *(a) TSW12*, *(b) CT208*, *(c) His1*, and *(d) TPP* below the diagonal and average pairwise F_{ST} of the reference genes above the diagonal. F_{ST} values are represented in colour from blue (F_{ST} = 0) to red (F_{ST} = 1). Populations are sorted as in figure S6.

References

1. The Tomato Genome Consortium. 2012 The tomato genome sequence provides insights into fleshy fruit evolution. *Nature* **485**, 635-641. (doi:10.1038/nature11119)
2. Shinozaki K, Yamaguchi-Shinozaki K. 2007 Gene networks involved in drought stress response and tolerance. *J. Exp. Bot.* **58**, 221-227. (doi:10.1093/jxb/erl164)
3. Yañez M, Cáceres S, Orellana S, Bastías A, Verdugo I, Ruiz-Lara S, Casaretto JA. 2009 An abiotic stress-responsive bZIP transcription factor from wild and cultivated tomatoes regulates stress-related genes. *Plant Cell. Rep.* **28**, 1497-1507. (doi:10.1007/s00299-009-0749-4)
4. Orellana S, Yañez M, Espinoza A, Verdugo I, González E, Ruiz-Lara S, Casaretto JA. 2010 The transcription factor SlAREB1 confers drought, salt stress tolerance and regulates biotic and abiotic stress-related genes in tomato. *Plant Cell Environ.* **33**, 2191-2208. (doi:10.1111/j.1365-3040.2010.02220.x)
5. Wu L, Chen X, Ren H, Zhang Z, Zhang H, Wang J, Wang XC, Huang R. 2007 ERF protein JERF1 that transcriptionally modulates the expression of abscisic acid biosynthesis-related gene enhances the tolerance under salinity and cold in tobacco. *Planta* **226**, 815-825. (doi:10.1007/s00425-007-0528-9)
